# Risk in the “Red Zone”: Outcomes for Children Admitted to Ebola Holding Units in Sierra Leone Without Ebola Virus Disease

**DOI:** 10.1093/cid/cix223

**Published:** 2017-03-20

**Authors:** F Fitzgerald, K Wing, A Naveed, M Gbessay, JCG Ross, F Checchi, D Youkee, MB Jalloh, D Baion, A Mustapha, H Jah, S Lako, S Oza, S Boufkhed, R Feury, J Bielicki, E Williamson, D M Gibb, N Klein, F Sahr, S Yeung

**Affiliations:** 1 Infection, Immunity, Inflammation and Physiological Medicine, UCL Great Ormond Street Institute of Child Health, United Kingdom;; 2 Save the Children, Sierra Leone and United Kingdom;; 3 Faculty of Epidemiology and Population Health, London School of Hygiene and Tropical Medicine, and; 4 Kings Sierra Leone Partnership, Kings Centre for Global Health, Kings College London, United Kingdom;; 5 34 Military Hospital, Republic of Sierra Leone Armed Forces, Freetown,; 6 Ola During Children’s Hospital, Sierra Leone Ministry of Health, Freetown,; 7 Cap Anamur (German Emergency Doctors), Ola During Children’s Hospital, Freetown, and; 8 Welbodi Partnership, Ola During Children’s Hospital, Freetown, Sierra Leone;; 9 Department of Health Services Research and Policy, London School of Hygiene and Tropical Medicine, United Kingdom;; 10 Western Area Emergency Response Centre, Freetown, Sierra Leone;; 11 MRC Clinical Trials Unit at UCL,; 12 Department of Medical Statistics, London School of Hygiene and Tropical Medicine,; 13 Farr Institute of Health Informatics, London, and; 14 Department of Clinical Research, London School of Hygiene and Tropical Medicine, United Kingdom

**Keywords:** Ebola virus disease, viral hemorrhagic fever, children, pediatrics, nosocomial infection

## Abstract

We collected data on 1054 children admitted to Ebola Holding Units in Sierra Leone and describe outcomes of 697/1054 children testing negative for Ebola virus disease (EVD) and accompanying caregivers. Case-fatality was 9%; 3/630 (0.5%) children discharged testing negative were readmitted EVD-positive. Nosocomial EVD transmission risk may be lower than feared.

The Ebola virus disease (EVD) outbreak in West Africa claimed >11 000 lives. Excess deaths from non-EVD conditions may have been far higher due to the impact of the outbreak on provision and use of healthcare [1]. Fear of nosocomial EVD infection may have delayed or prevented attendance for routine and emergency care: the rate of avoidable all-cause mortality was estimated as three times higher than nonoutbreak periods [1]. In the Western Area of Sierra Leone patients with suspected EVD were isolated in “Red Zones” in Ebola holding units (EHUs) while awaiting results of EVD tests [2]. Turnaround times for results averaged 48 hours but were frequently longer, due to overwhelming demand and system bottlenecks [2, 3]. Concerns were raised that EHUs could act as amplification sites for EVD owing to close proximity between patients and duration of exposure [4, 5]. The screening criteria for suspect EVD were broad, particularly for children (Supplementary Appendix Figure 1) [6]. Only 1 symptom and fever was sufficient, versus fever and 3 symptoms in adults [1, 6]. Children were challenging to care for, both clinically and to provide adequate supervision to minimize cross-infection as many were admitted alone without a caregiver [2]. It was difficult to ensure ambulant toddlers, and children stayed in their bedspace and did not touch potentially infectious items such as latrine buckets. Therefore, not only did children have a lower threshold for admission compared to adults but also higher risk of exposure to nosocomial infection.

Standard care included antibiotics, antimalarials, and symptomatic treatment, but the level of intervention varied by site and resource availability [2]. The structure of EHUs also varied: some were purpose-built isolation units, but most were converted wards or tents onsite with existing health facilities. Many EHUs could not cohort patients by risk into suspect/probable bays due to lack of space; and most had buckets/chair-latrines by each bedspace although some facilities had shared latrines. Figure 1 is a representation of an EHU with possible sites of cross-infection marked.

**Figure 1.**
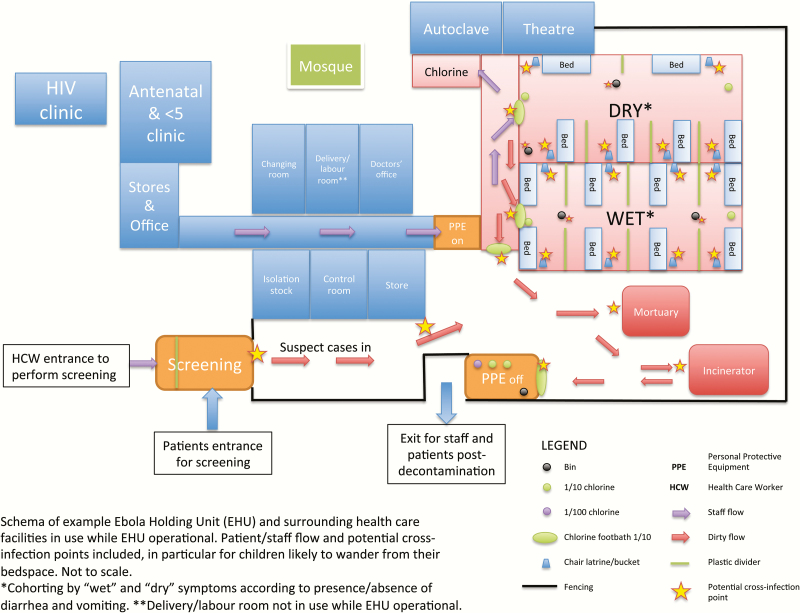
Schema of example Ebola holding unit (EHU) and surrounding healthcare facilities in use while EHU was operational. Patient/staff flow and potential cross-infection points included, in particular for children likely to wander from their bedspace.

On receiving EVD-negative test results, children requiring further clinical care were admitted to the general ward of Ola During Children’s Hospital (ODCH), the main children’s hospital. The remainder were discharged home or to an observational interim care center (OICC), where unaccompanied high-risk children were observed for the 21-day incubation period.

Our objective in this study was to describe outcomes in EVD-negative children admitted to EHUs and to estimate rates of readmission with EVD to quantify nosocomial infection risk.

## METHODS

All children <13 years of age presenting to 11 EHUs in the Western Area, Sierra Leone between August 14, 2014, and March 31, 2015, were eligible for inclusion in this retrospective cohort study.

Settings and data collection methods have been described previously [2]. Each EHU was visited to extract data from paper clinical records, case investigation forms, and site admission books, and to conduct staff interviews (Supplementary Appendix 1a). Data were obtained from clinical records at ODCH on EVD-negative children after transfer from EHUs. Data were cross-referenced with the Western Area Ebola Response Centre (WAERC) database (which contained demographic information used for coordinating bed management), burial records, child protection records, and laboratory results. Data were single-entered directly into a password-protected database (EpiInfo™ v7.1.4). A record-matching schema was developed to identify children who were readmitted to EHUs having previously been discharged with a negative test or died at home with an EVD-positive swab result. (Supplementary Appendix 1b) [2]. Telephone calls were made to guardians of EVD-negative children ≥3 weeks postdischarge to check the child’s health and that of guardians who accompanied their children into EHUs as asymptomatic caregivers and therefore also were at risk of nosocomial EVD infection.

Analyses were performed using STATA (v14.0). The proportion of suspect cases that tested negative (overall and by month) was calculated. Further analyses were limited to children testing EVD-negative with an ascertained mortality outcome and included calculation of case fatality ratio (including those who died in the EHU or after transfer to ODCH), rate of readmission with a positive EVD test, and caregiver nosocomial infection attack rates (Supplementary Appendix 1c).

Approval was obtained from the Sierra Leone Ethics and Scientific Review Committee and the London School of Hygiene and Tropical Medicine Ethics committee (ref 8924).

## RESULTS

Overall, 1054 children were admitted to 11 EHUs in the Western Area between August 2014 and March 2015. Admissions per week rose from a median (interquartile range [IQR]) of 8 (5–11) (August–October 2014) to 50 (40–58) (February–March 2015) (Supplementary Appendix Figure 2). Of 1054 children, 697 (69%) tested EVD-negative; with known outcomes for 696 (99.9%). The proportion of those testing negative increased from 23% (95% confidence interval [CI], 12–38%) (October 2014) to 94% (95% CI, 89%–96%) (February 2015) (Supplementary Appendix Figure 2).

Of the 696 EVD-negative children with known outcomes, median age was 3 years, (IQR, 1–7), and 50% were female. Of children for whom data were available, 105/621 (17%) were admitted unaccompanied, and EVD contact was reported in 108/541 (20%) (Supplementary Appendix Table 1). Median time from symptom onset to presentation was 2 days (IQR, 1–3). Antibiotics were received by 407/494 (82%), antimalarials by 416/494 (84%), and intravenous fluids by 101/265 (38%) (Supplementary Appendix Table 1). The case-fatality ratio (CFR) was 9% (66/696, 95% CI 8%–12%). Of the 638 (92%) children surviving to EHU discharge, 120 (19%) were admitted to ODCH, where 8 (1%) more children died; 464 (73%) were discharged home, 12 to an OICC (2%), and 6 to an orphanage (1%). For 36 (6%) the discharge location was unknown.

Of the 630 children who survived, only 3 (0.5%) were subsequently readmitted within 21 days and tested EVD-positive. All 3 had a parent with EVD documented at first admission. Of the 483 caregivers admitted with EVD negative children who survived to discharge, 105 (22%) were contactable. None had been admitted with EVD. The caregivers contacted were exposed to similar proxy risk factors to those not contacted (Supplementary Appendix Table 2).

## DISCUSSION

During the EVD outbreak, fear of nosocomial infection severely impacted on the provision and utilization of healthcare [1, 7]. It was difficult to provide children with adequate care and supervision in the EHUs, especially those who were admitted unaccompanied. Given the low threshold for isolating children with suspected EVD infection, a substantial number of uninfected children were exposed to these risks. To date, there has been little evidence to address these concerns. The data from this large multicenter study are potentially reassuring.

The 9% CFR we report in EVD-negative children is in line with the CFR (10–12%) for inpatients admitted to the children’s hospital prior to the epidemic and in other African inpatient settings (5–20%) [8, 9] but higher than would be expected from primary health facilities. Interpretation of the CFR is not straightforward. The EVD-negative cohort comprised both sick children who would normally be admitted as inpatients and less sick children who would normally attend primary healthcare facilities. Given the reductions in healthcare utilization during the epidemic those who did attend may have been sicker than in nonepidemic times [1]. This reduction in admissions is seen in Supplementary Appendix Figure 2: only by February/March 2015 did admissions approximate usual weekly rates at ODCH.

We report a low rate of nosocomial infections. Only 3/630 (0.5%) of surviving EVD-negative children were subsequently readmitted with EVD, all of whom had a parent with confirmed EVD prior to first admission. These results support 2 other studies that demonstrate low readmission rates in mixed age populations (3.3–7%) [5, 10]. Additionally, of the asymptomatic caregivers who had accompanied EVD-negative children into “red zones” and whom we managed to contact, none were subsequently admitted themselves with EVD. There are a number of possible explanations for these results. The majority (59%) of this cohort were admitted to the paediatric EHU built onsite at ODCH, which was spacious (≥2.5 m between beds). Strict advice was given to guardians about hand hygiene and keeping within their bed-space. Previous evidence suggests that it is the direct exposure to bodily fluids and the sharing of latrine facilities which increase infection risk, rather than sharing a ward per se, so infants in nappies may have experienced less risk [7]. Furthermore, it appears that children may be less susceptible than adults to EVD infection, although the mechanism underlying this is undetermined [11]. Applicability of these results to adult populations in EHUs is unclear. Children might be more exposed owing to the challenges of maintaining patient separation, but data from mixed-age populations showed a slightly higher rate of positive readmissions [5, 10]. Although lack of evidence of caregiver nosocomial infection is partly reassuring, asymptomatic caregivers may differ significantly from unwell adults admitted to EHUs for testing.

As with all studies from emergency settings, where missing and unreliable data are a key limitation, these results must be interpreted cautiously. Substantial effort was made to check for potential paediatric readmissions across 11 EHUs (representing the bulk of the Western Area), and district laboratory results (which should have captured children dying at home with an EVD-positive mouth swab result). However, some readmissions/deaths may have been missed due to discrepancies in name spelling or age reporting, and children dying at home unreported or in a different district [12]. Additionally, a minority of caregivers were contacted: some caregiver nosocomial infections may have been missed.

In conclusion, our results suggest that nosocomial transmission of EVD in children and their caregivers in Western Sierra Leone may be lower than feared. This is testament to the dedication of those managing EHUs to ensure infection control procedures and treating children under extremely challenging circumstances.

## Supplementary Data

Supplementary materials are available at *Clinical Infectious Diseases* online. Consisting of data provided by the authors to benefit the reader, the posted materials are not copyedited and are the sole responsibility of the authors, so questions or comments should be addressed to the corresponding author.

## Supplementary Material

Supplementary_Figure_1

Supplementary_Figure_2

Supplementary_Table_1

Supplementary_Table_2

Supplementary_Material

## Notes

***Author Contributions.*** Concept and study design by F. F., K. W., F. C., J. B., D. M. G., N. K., F. S., and S. Y. with input from other authors. Data collected by F. F., A. N., M. G., J. C. G., D. Y., M. J., D. B., A. M., H. J., and R. F. Data management, cleaning, and analysis performed by K. W., S. O., S. B., with contributions from E. W., F. F., F. C., and S. Y. Manuscript drafted by F. F. and K. W. with input from S. Y. and D. M. G. All authors proofread and approved final draft.

***Acknowledgments.*** We are grateful to the following organizations and individuals for their assistance and sharing data: Dr. T. B. Kamara, Medical Director, Connaught Hospital, Freetown, Sierra Leone; Dr. B. E. Parker, Medical Superintendent, Macauley St Hospital, Freetown, Sierra Leone; Dr. V. George, Medical Superintendent, Lumley Government Hospital, Freetown, Sierra Leone; Dr. King, Medical Superintendent, Rokupa Government Hospital, Freetown, Sierra Leone; Ms. F. Koroma, Community Health Officer, Newton Community Health Centre, Western Area Rural, Sierra Leone; Mrs. Z. M. Cooper, Western Area Coordinator, Rural, Sierra Leone; Mr. I. Sesay, Western Area Coordinator, Urban, Sierra Leone; the Live Case Management Team, Western Area Emergency Response Center, Sierra Leone; Ms. Marion Dumbuya, 34 Military Hospital, Freetown, Sierra Leone; Dr. Brima Kargbo, Chief Medical Officer, Ministry of Health, Sierra Leone; Dr. Marta Lado and Mr. Quaanan Kessete, Kings Sierra Leone Partnership, Freetown, Sierra Leone; Dr. Iza Ciglenecki and Ms. Monia Sayah, Médecins sans Frontières Switzerland; Dr. Rupert Gould, International Federation of Red Cross and Red Crescent Societies; Dr. Brian Raleigh, Aspen Medical; Dr. Trina Helderman, Dr. David Sinclair, MEDAIR; Dr. Hana Rohan, E-health Africa; Dr. Tim Brooks, Public Health England; Miss Charity Garnett, NHS England; Miss Rachael Cummings and Miss Kate Jarman, Save the Children International, as well as the nurses, cleaners and community health officers at each facility.

***Funding.*** This work was supported by Save the Children. F. F. is supported by a grant from the Medical Research Council (MR/K023535/1) and the National Institute for Health Research Biomedical Research Centre at Great Ormond Street Hospital for Children NHS Foundation Trust and University College London. S. Y. was supported by a Wellcome Trust Institutional Strategic Support Fund awarded to the London School of Hygiene and Tropical Medicine.

***Potential Conflicts of Interest.*** The authors declare no conflicts of interest but would like to disclose the following: J. B. has disclosed the following relevant financial relationships: other (spouse) is Senior Legal Counsel at Novartis and owns stocks and stock options from Novartis. D. M. G. has disclosed the following relevant financial relationships: served as an advisor or consultant for ViiV Healthcare Cabotegravir Advisory Committee. The authors have submitted the ICMJE Form for Disclosure of Potential Conflicts of Interest. Conflicts that the editors consider relevant to the content of the manuscript have been disclosed.
