# Supplementary figures and images for: Risk in the “Red Zone”: Outcomes for Children Admitted to Ebola Holding Units in Sierra Leone Without Ebola Virus Disease
Source: Clin Infect Dis. 2017 Mar 20;65(1):162–5. doi: 10.1093/cid/cix223 (PMC5693324; doi:10.1093/cid/cix223)

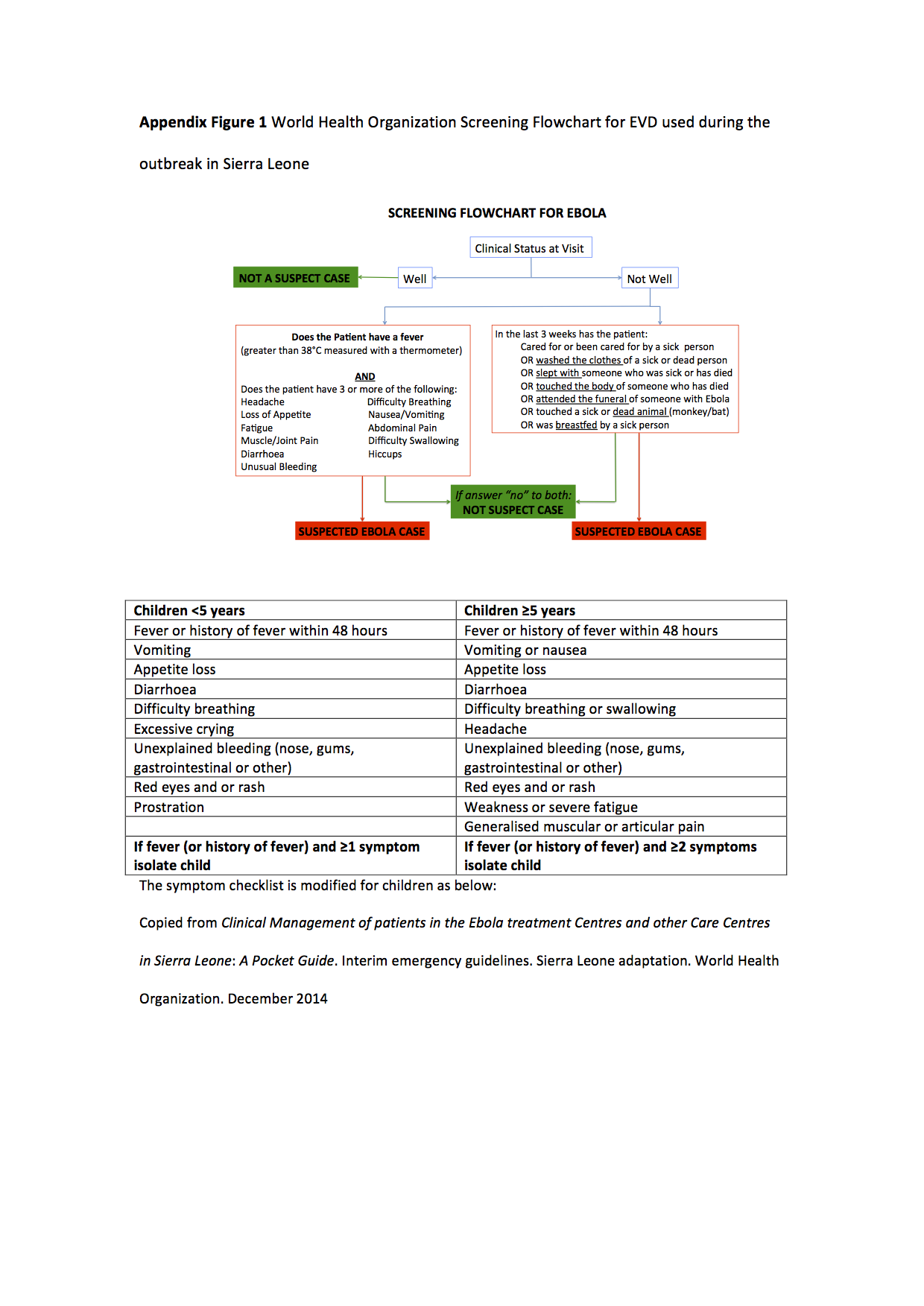

Supplement: Supplementary_Figure_1 [file cix223_suppl_supplementary_figure_1.png]

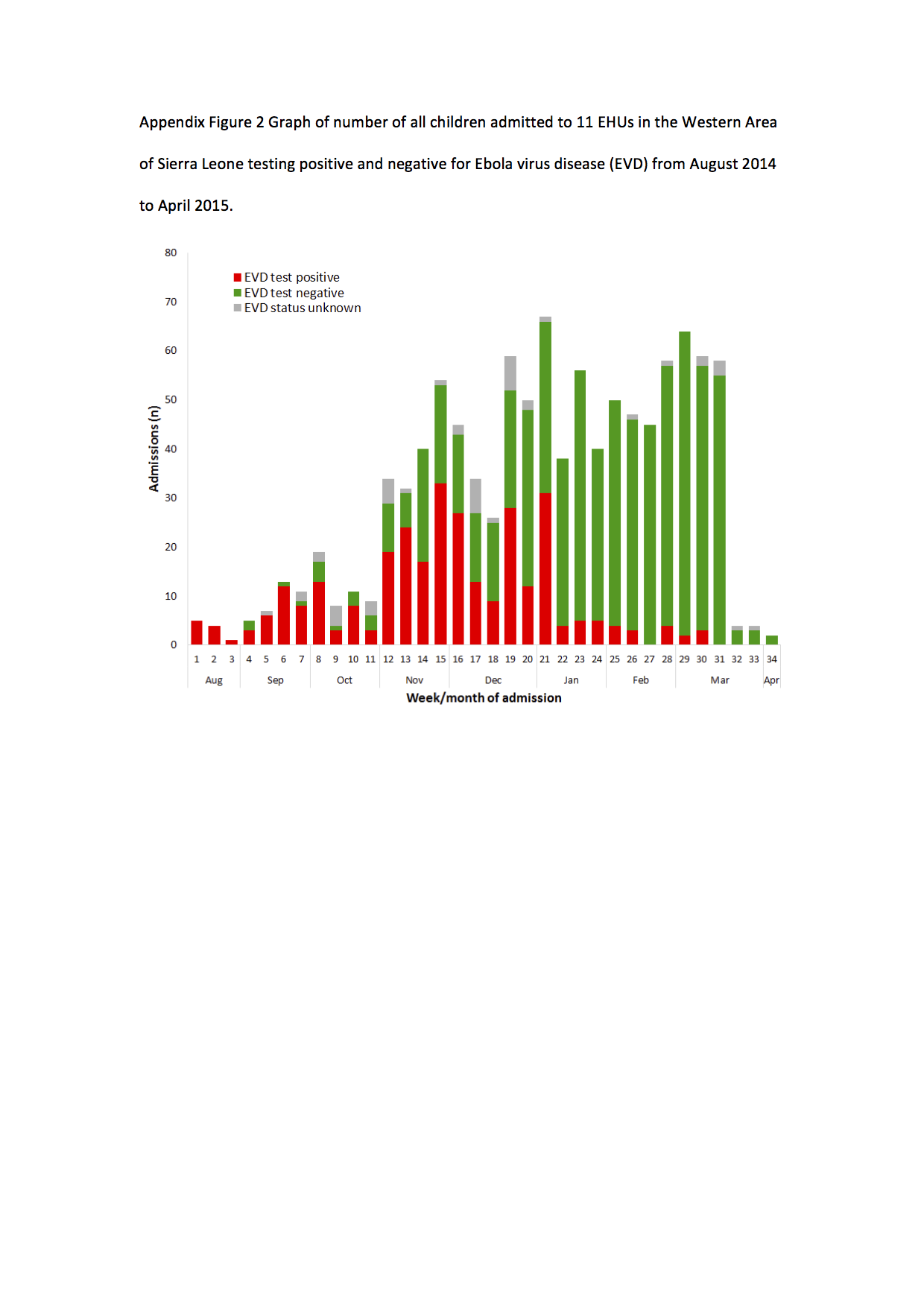

Supplement: Supplementary_Figure_2 [file cix223_suppl_supplementary_figure_2.png]
