# Supplementary material for: Risk in the “Red Zone”: Outcomes for Children Admitted to Ebola Holding Units in Sierra Leone Without Ebola Virus Disease
Source: Clin Infect Dis. 2017 Mar 20;65(1):162–5. doi: 10.1093/cid/cix223 (PMC5693324; doi:10.1093/cid/cix223)
Supplement: Supplementary_Table_1 [file cix223_suppl_supplementary_table_1.docx]

**Appendix Table 1: Characteristics of all (697) children who attended an Ebola holding unit (EHU), had EVD test results recorded, and had a final negative EVD test result**

|  |  | |  |
| --- | --- | --- | --- |
|  | n | % (95% CI) |  |
| **Total^1^** | 697 | 100 |  |
|  |  |  |  |
| **Female** | 348 | 50 (46-54) |  |
|  |  |  |  |
| **Median age (years (IQR))** | 3 (1 - 7) | - |  |
|  |  |  |  |
| **Age 0 – 2 yrs** | 336 | 48 (44-52) |  |
|  |  |  |  |
| **Positive contact** (n^2^=541) | 108 | 20 (17-24) |  |
|  |  |  |  |
| **Median days (IQR) symptoms to HU admission** (n=772) | 2 (1 - 3) | - |  |
|  |  |  |  |
| **Admitted with caregiver** (n=621) | 516 | 83 (80-86) |  |
|  |  |  |  |
| **Fever^3^** (n=566) | 528 | 93 (91-95) |  |
|  |  |  |  |
| **Fatigue/weakness** (n=407) | 393 | 97 (94-98) |  |
|  |  |  |  |
| **Vomiting/nausea** (n=556) | 345 | 62 (58-66) |  |
|  |  |  |  |
| **Diarrhoea** (n=548) | 252 | 46 (42-50) |  |
|  |  |  |  |
| **Conjunctivitis** (n=463) | 73 | 16 (13-19) |  |
|  |  |  |  |
| **Anorexia** (n=560) | 452 | 81 (77-84) |  |
|  |  |  |  |
| **Abdominal pain** (n=392) | 155 | 40 (35-45) |  |
|  |  |  |  |
| **Muscle pain** (n=377) | 127 | 34 (29-39) |  |
|  |  |  |  |
| **Joint pain** (n=368) | 102 | 28 (23-33) |  |
|  |  |  |  |
| **Headache** (n=397) | 256 | 65 (60-69) |  |
|  |  |  |  |
| **Difficulty breathing** (n=533) | 169 | 32 (28-36) |  |
|  |  |  |  |
| **Difficulty swallowing** (n=481) | 130 | 27 (23-31) |  |
|  |  |  |  |
| **Skin rash** (n=522) | 88 | 17 (14-20) |  |
|  |  |  |  |
| **Cough** (n=407) | 57 | 14 (11-18) |  |
|  |  |  |  |
| **Hiccups** (n=519) | 52 | 10 (8-13) |  |
|  |  |  |  |
| **Unexplained bleeding** (n=518) | 19 | 4 (2-6) |  |
|  |  |  |  |
| **Received antibiotic^4^** (n=494) | 407 | 82 (79-86) |  |
|  |  |  |  |
| **Received antimalarial^4^** (n=494) | 416 | 84 (81-87) |  |
|  |  |  |  |
| **Received IV treatment^4^** (n=265) | 101 | 38 (32-44) |  |
|  |  |  |  |
| **Malaria RDT+** (n=57) | 31 | 54 (41-68) |  |
|  |  |  |  |
| **Median days (IQR) of EHU stay^5^** | 2 (1 - 2) | - |  |
| **Note 1:** Total = total children admitted to holding units with test results available who tested negative for EVD. **Note 2:** n=number of (EVD-negative) children with recorded data for variable. **Note 3:** All symptoms in this table: recorded upon presentation at holding unit (EHU). **Note 4:** At holding unit. **Note 5:** Time from EHU admission until death, discharge or transfer. | | | |
|  |  |  |  |
