# Supplementary material for: Risk in the “Red Zone”: Outcomes for Children Admitted to Ebola Holding Units in Sierra Leone Without Ebola Virus Disease
Source: Clin Infect Dis. 2017 Mar 20;65(1):162–5. doi: 10.1093/cid/cix223 (PMC5693324; doi:10.1093/cid/cix223)
Supplement: Supplementary_Table_2 [file cix223_suppl_supplementary_table_2.docx]

**Appendix Table 2: Distribution of potential risk factors for caregivers admitted with 483 children who attended a holding unit (HU), had a negative EVD test result recorded, were admitted with a caregiver, survived to be discharged by (1) whether or not valid contact details were provided and (2) whether or not they were successfully contacted after discharge.**

|  |  | **Total (N=483)** |  | **No contact****details (N=269)** |  | **Contact details****provided (N=214)** | | |  |  |
| --- | --- | --- | --- | --- | --- | --- | --- | --- | --- | --- |
|  |  |  |  |  |  | Not contacted | Contacted |  |  |  |
|  |  | n (%) |  | n (%) |  | n (%) | n (%) |  |  |  |
| **Total^1^** | All children | 483 (100) |  | 269 (56) |  | 109 (23) | 105 (22) | | |  |
|  |  |  |  |  |  |  |  | | |  |
| **Holding unit** | ODCH | 410 (85) |  | 232 (86) |  | 87 (80) | 91 (87) | | |  |
|  | Connaught | 7 (1) |  | 4 (1) |  | 3 (3) | 0 (0) | | |  |
|  | Lumley | 10 (2) |  | 7 (3) |  | 2 (2) | 1 (1) | | |  |
|  | Rokupa | 8 (2) |  | 6 (2) |  | 1 (1) | 1 (1) | | |  |
|  | Macauley | 5 (1) |  | 2 (1) |  | 1 (1) | 2 (2) | | |  |
|  | Newton | 9 (2) |  | 3 (1) |  | 2 (2) | 4 (4) | | |  |
|  | Kerry town | 9 (2) |  | 4 (1) |  | 5 (5) | 0 (0) | | |  |
|  | PTS2 | 23 (5) |  | 9 (3) |  | 8 (7) | 6 (6) | | |  |
|  | Aspen | 2 (0) |  | 2 (1) |  | 0 (0) | 0 (0) | | |  |
|  |  |  |  |  |  |  |  | | |  |
| **Month of admission** | Sep | 1 (0) |  | 1 (0) |  | 0 (0) | 0 (0) | | |  |
|  | Oct | 8 (2) |  | 6 (2) |  | 0 (0) | 2 (2) | | |  |
|  | Nov | 42 (9) |  | 22 (8) |  | 14 (13) | 6 (6) | | |  |
|  | Dec | 57 (12) |  | 37 (14) |  | 6 (6) | 14 (13) | | |  |
|  | Jan | 112 (23) |  | 62 (23) |  | 29 (27) | 21 (20) | | |  |
|  | Feb | 140 (29) |  | 52 (19) |  | 40 (37) | 48 (46) | | |  |
|  | Mar | 122 (25) |  | 88 (33) |  | 20 (18) | 14 (13) | | |  |
|  |  |  |  |  |  |  |  | | |  |
| **Duration of EHU stay^2^** (n^3^=213) | Median (IQR) | 2 (1-2) |  | 2 (1-2) |  | 2 (1-2) | 2 (1-2) | | |  |
|  |  |  |  |  |  |  |  |  |  |  |
| **% EVD-positive admitted^4^** | Median (IQR) | 6.9 (3.1-24) |  | 6.9 (3.1-24) |  | 6.9 (3.1-12.5) | 6.9 (3.1-12.5) | | | |
| **Note 1:** Total = total children admitted to holding units. **Note 2:** Time from admission EHU admission until discharge or transfer. **Note 3:** n=number of children with recorded data for variable. **Note 4:** % of children testing EVD-positive during week of admission | | | | | | | | | | |
