# Supplementary material for: Risk in the “Red Zone”: Outcomes for Children Admitted to Ebola Holding Units in Sierra Leone Without Ebola Virus Disease
Source: Clin Infect Dis. 2017 Mar 20;65(1):162–5. doi: 10.1093/cid/cix223 (PMC5693324; doi:10.1093/cid/cix223)
Supplement: Supplementary_Material [file cix223_suppl_supplementary_material.docx]

**Supplementary Appendix**

**Contents**

1. **Methods**
   1. **Data Collection**
   2. **Patient Matching Schema**
   3. **Statistical analysis**
2. **Appendix Table 1:** Characteristics of all (697) children who attended an Ebola holding unit (EHU), had EVD test results recorded, and had a final negative EVD test result
3. **Appendix Table 2:** Distribution of potential risk factors for caregivers admitted with 483 children who attended a holding unit (HU), had a negative EVD test result recorded, were admitted with a caregiver, survived to be discharged by (1) whether or not valid contact details were provided and (2) whether or not they were successfully contacted after discharge.
4. **Appendix Figure 1:** Screening Flowchart for EVD used at Ola During Children’s Hospital, (ODCH) in Freetown throughout the outbreak and incorporated into World Health Organisation guidelines from November 2014
5. **Appendix Figure 2:** Graph of number of all children admitted to 11 EHUs in the Western Area of Sierra Leone testing positive and negative for Ebola virus disease (EVD) from August 2014 to April 2015.
6. **Methods**
   1. **Data Collection**

Site visits were carried out by the lead investigator (FF) to collect data from case investigation forms, site admission books, and other clinical records (e.g. hospital records at Ola During Children’s Hospital) and carry out staff interviews. Data were cross referenced with the Western Area Emergency Response Centre (WAERC) database which held demographic information used for coordinating bed management and transfers; district-wide laboratory results; child protection registers; burial records, and the database of the emergency telephone service for ambulance notification. Single data entry was carried out by FF and AN for expediency.

- 1. **Patient Matching Schema**

As previously described^1^, a complete match consisted of the criteria below:

- Matching Western Urban Area (WUR) number and matching name. The WUR number was allocated with each case investigation form but was used inconsistently.
- Matching name, age & case investigation (CI) form date
- Four or more of name, age, case investigation form/laboratory test date, address, holding unit, eventual status (positive/negative/transferred/discharged)

A partial match consisted of 3 or more of name, age, CI form date, address, holding unit, eventual status (positive/negative/transferred/discharged). For identifying subsequent readmissions, a second CI form or laboratory test result date within 21 days of the first (the incubation period for EVD) were included as criteria, as were positive identifications by site staff during interview or documentation in site admission books of a readmission. Small discrepancies in name spelling (e.g. Mohammed and Mohamed) could still be included as a complete match, but larger discrepancies of several letters (e.g. Abu and Abubakar) were a partial match. Matching was performed by 2 investigators (MG and JCG). Any discrepancies between the 2 investigators’ categorization were raised with the lead investigator (FF) with whom the final decision rested. Partial matches were reviewed by the lead investigator and either discarded or included depending on any additional information available (e.g. from telephone follow ups). All complete matches were included in the analysis.

- 1. **Statistical analysis**

All personal identifiers were removed prior to analysis. Characteristics compared between the children of contactable and uncontactable caregivers (both those without contact details and those with details but uncontactable) as proxies of caregiver infection risk were: month of admission (a proxy for risk of EVD exposure in an EHU as proportions of those testing positive and negative changed over time^5^), percentage of children testing positive during the week of admission (assessing exposure risk), EHU attended and duration of admission.

References

1. Fitzgerald F., Naveed A., Wing K., Gbessay M., Ross J.C.G., Checchi F., Youkee D., Jalloh M., Baion D., Mustapha A., Jah H., Lako S., Oza S., Boufkhed S., Feury R., Bielicki J., Gibb D., Klein N., Sahr F., Yeung S. Ebola Virus Disease in children in Sierra Leone: a retrospective cohort study. *Emerging infectious diseases* 2016.

2. Brown C, Kessete Q., Baker P., Youkee D., Walker N., Kamara T.B., Kamboz A., Johnson O., Lado M. Bottlenecks in health systems functioning for control of Ebola virus disease in Connaught Hospital, Freetown, Sierra Leone. 26th European Congress of Clinical Microbiology and Infectious Diseases. Amsterdam; 2016. p. E poster P0092.
